# Intelligent Techniques Using Molecular Data Analysis in Leukaemia: An Opportunity for Personalized Medicine Support System

**DOI:** 10.1155/2017/3587309

**Published:** 2017-07-25

**Authors:** Haneen Banjar, David Adelson, Fred Brown, Naeem Chaudhri

**Affiliations:** ^1^School of Computer Science, University of Adelaide, Adelaide, SA, Australia; ^2^Department of Computer Science, King Abdulaziz University, Jeddah, Saudi Arabia; ^3^School of Molecular and Biomedical Science, University of Adelaide, Adelaide, SA, Australia; ^4^Oncology Centre, Section of Hematology, HSCT, King Faisal Specialist Hospital and Research Centre, Riyadh, Saudi Arabia

## Abstract

The use of intelligent techniques in medicine has brought a ray of hope in terms of treating leukaemia patients. Personalized treatment uses patient's genetic profile to select a mode of treatment. This process makes use of molecular technology and machine learning, to determine the most suitable approach to treating a leukaemia patient. Until now, no reviews have been published from a computational perspective concerning the development of personalized medicine intelligent techniques for leukaemia patients using molecular data analysis. This review studies the published empirical research on personalized medicine in leukaemia and synthesizes findings across studies related to intelligence techniques in leukaemia, with specific attention to particular categories of these studies to help identify opportunities for further research into personalized medicine support systems in chronic myeloid leukaemia. A systematic search was carried out to identify studies using intelligence techniques in leukaemia and to categorize these studies based on leukaemia type and also the task, data source, and purpose of the studies. Most studies used molecular data analysis for personalized medicine, but future advancement for leukaemia patients requires molecular models that use advanced machine-learning methods to automate decision-making in treatment management to deliver supportive medical information to the patient in clinical practice.

## 1. Introduction

Molecular cytogenetics of hematological malignancies and therapies is under development. Leukaemia is a hematological disorder where two leukaemia patients who may appear identical morphologically may have different molecular profiles and thus the variation in response to the prescribed therapies would be unpredictable [1]. Leukaemia usually begins in the bone marrow, which is the site where all blood cells are formed and produced. When a person has leukaemia, the white blood cells produced are usually numerous, and they are abnormal, which means that they cannot effectively defend the body from diseases, pathogens, or foreign substances. The type of white blood cells affected, either lymphoid or myeloid, can identify the type of leukaemia. Four common types of leukaemia are chronic lymphocytic leukaemia (CLL), chronic myeloid leukaemia (CML), acute lymphocytic leukaemia (ALL), and acute myeloid leukaemia (AML) [2].

The most common modes of treatment for leukaemia involve chemotherapy, radiation therapy, stem cell transplantation, and immunotherapy with interferon [2]. Many patients become disease-free after years of treatment, proceeding to live normal lives. However, these modes of therapy can have disastrous consequences for the victims of leukaemia. For instance, chemotherapy often makes patients lose their hair, it can darken their skin, and it can cause infertility in young patients. Bone marrow transplantation is also very expensive, and it is not often easy to find a matching bone marrow donor, especially if close family members are not a match. The pharmacogenomics (PGx) aims to replace general modes of treatment, such as chemotherapy and bone marrow transplantation, adopting instead a tailor-made course of medication designed according to a patient's particular genetic makeup [3]. Although multiple targeted therapies are available to use for leukaemia patients [4], it is difficult to select among the available targeted therapies.

Therefore, the use of intelligent techniques in medicine has brought a ray of hope in terms of treating leukaemia patients. Intelligent techniques are able to conduct automatic searches to discover knowledge and learn from data to facilitate the task and achieve the objective. The broad areas frequently defined under intelligence techniques are as follows: knowledge discovery, machine learning, and data mining. These areas use statistics and probability to detect patterns that are difficult to study manually. Intelligent techniques will integrate various molecular technologies and sources of data, information, or knowledge to facilitate the development of personalized medicine and decision-making by physicians.

The personalized decision support system requires personal information or genetic information, such as genetic tests and medical tests, for each patient to integrate, as far as possible, the knowledge gained from genomics research relating to the disease in question [5]. From this definition, it is clear that the need for personalized decision support systems in leukaemia treatment has increased because of the massive amount of available genetic and genomic information. With the use of the personalized medicine support system, leukaemia treatment will no longer be a trial and error game and it will be possible to select which drugs will work at which stage. The outcomes are expected to provide a preventive, next-generation therapy, with better specific interventions for individual patients.

Personalized medicine support systems can use available knowledge resources to deliver just-in-time information to individualize therapy. The existing pharmacogenomics knowledge base (PharmGKB) (available at http://www.pharmgkb.org) is a massive resource that provides researchers with information relevant to genetic variations on drug responses. The second source is to translate PGx knowledge into a rule-based representation where the rules are extracted from the characteristics of PGx knowledge in the US Food and Drug Administration (FDA) drug label database [6]. Another knowledge resource is the Clinical Practice Guidelines (CPG) document, which lists a set of guidelines for cases with specific diagnoses, along with recommended therapeutic action plans [7].

Developing personalized medicine support systems in some medical applications has already made significant progress. First, in cardiovascular diseases, many factors could influence cardiovascular disease, such as genes, environment, and lifestyle (exercise and nutrition). It was important to develop models for prevention, treatment management, or detecting disease to assist clinicians in treating cardiovascular patients. Indeed, the personalized decision support system for cardiovascular patients was constructed using two models. One model was for risk assessment using patients' personal information, and the other was for generating advice to clinicians based on the first model's results [8]. The second application was in type 1 diabetes mellitus. The frequent measurements of glucose levels, monitoring physical activity, and personal information about the genome, such as the genes that could cause obesity and predisposition for diabetes, were used to create a personalized decision support system for treating, preventing, and monitoring patients [9]. Another system for optimizing insulin infusion rates based on nonlinear model predictive control was designed using in silico data from a virtual type 1 diabetes patient, where the system was evaluated using data from a mathematical model of a patient with type 1 diabetes. The results showed the effectiveness of using such methods with diabetes patients [10]. The third application is colon cancer, where selection of the treatment plan was the objective of the personalized medicine support system. The clinical and genomic features were used for early diagnosis in colon cancer using cluster techniques. The platform known as MATCH supported clinicians in making decisions about patients with colon cancer [11]. Adding genetic features improves diagnosis compared to previous research methods. Further methods proposed for colon cancer prediction based on genetic information were studied by Kulkarni et al. [12]. The last example, but not limited to those mentioned in our review, breast cancer classification is an active research application in which the personalized medicine support medicine was the objective in multiple studies [13–31].

One angle of personalized medicine is to identify the correct disease subtype and patient classification. Machine-learning techniques were proven to achieve a high performance classification in identifying patient subtypes by using a support vector machine (SVM) and uncertainty SVM [32], in predicting drug sensitivity in cancer by using Bayesian networks [33, 34], in predicting patient response to therapy by using ensemble methods [13], in predicting risk category using soft computing methods [35], or in recommendations to enhance lifestyle and educate patients about healthy solutions [36]. Thus, intelligent methods should be proposed to create a personalized medicine support system in leukaemia. These methods required information about preknowledge in allocating optimal treatment, responding to each patient's risk category, which should be provided to evaluate the models.

Medical researchers continue to emphasise that their studies are updated with the most effective treatment protocols, which could be used to treat leukaemia patients. The current system for achieving personalized medicine in leukaemia has been established by using the predictive factors to determine upfront treatment. Many groups of researchers have conducted studies by using different techniques to investigate several factors that could affect the drug responses. Studying a single biomarker as a predictive substance could indicate the response pretreatment and predict the risk to the individual [37]. The other approach involves predicting the drug reaction in terms of toxicity or resistance, using an individual's genotype data and clinical data to improve the individual's care [38]. A pharmacogenetics is a field that studies the individual's response to a specific therapy based on the person's genotype information. With the human molecular tests and the development of molecularly targeted therapy, the system for achieving personalized medicine for leukaemia has additional levels of information that needs to be processed with assistance from information technology.

According to current knowledge, many leukaemia researchers have applied intelligent techniques, but no reviewers have yet undertaken a systematized literature review from a computational perspective concerning the development of personalized medicine intelligent techniques for leukaemia patients using molecular data analysis. This review studies the published empirical research on personalized medicine in leukaemia and synthesizes findings across studies related to intelligence techniques in leukaemia, with specific attention to particular categories of these studies to help identify opportunities for further research into personalized medicine support systems in one category of leukaemia, namely, chronic myeloid leukaemia. A systematic search was carried out to identify studies using intelligence techniques in leukaemia and to categorize these studies based on leukaemia type and also the task, data source, and purpose of the studies. Our review is conducted to support health informatics and biomedical and bioinformatics in order to answer specific technical questions to help develop future research into leukaemia from a technical perspective.

## 2. Methods

### 2.1. Search Strategy

Ten databases were searched, including Scopus, PubMed, Web of Science, BIOSIS, Inspec, MEDLINE, Embase, Springer, ACM Digital Library, and IEEE Xplore. The review was restricted to English-language studies published from 2001 to October 2016 because, prior to 2001, molecular targeted therapies and molecular responses for personalized medicine were not approved by the FDA for medical treatments in leukaemia but became more popular around this time [39]. The search was limited to primary research articles. The eligibility of each study was evaluated based on the title and abstract. Only full-text articles were retrieved. The terms searched were leukaemia and leuk^*∗*^, with different combinations of key words for intelligent systems and techniques (machine learning, data mining, knowledge extraction, and CDS system) and with combined keywords and/or subject headings to identify technical leukaemia articles. Since studies were screened by a single researcher and then reviewed by the coauthors, this work cannot be considered a systematic review, but it could be considered a “systematized review” [40] to demonstrate comprehensive search guidelines and an elaborative quality assessment and synthesis of research evidence.

### 2.2. Selection of Studies and Data Extraction

The resulting abstracts were evaluated for inclusion. Then, the full text of those identified as meeting the criteria were obtained. Studies were included in the review, if the study

- used molecular data from adult leukaemia patients;
- used intelligence techniques to achieve the purpose of the study;
- was implemented as a model for adult leukaemia patients;
- was published in a peer-reviewed journal between 2001 and 2016;
- was published in English.

Because the intention was to review the literature to identify whether opportunities currently under clinical development are related to model analysis molecular data for personalized medicine in leukaemia, articles were excluded, if they

1. published decision-analytic models for economic purposes;
2. used pediatric leukaemia data;
3. studied a nonpatient population;
4. were not written in English;
5. were doctoral dissertations or pilot studies;
6. did not include the full text of the study report.

## 3. Results

### 3.1. Study Selection

**Figure 1:**
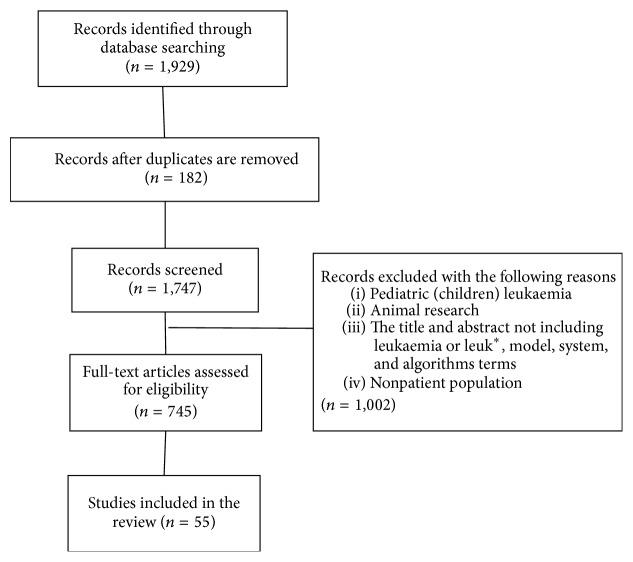
Flow chart showing the article-selection process.

In total, *n* = 1,929 citations were retrieved. Excluding duplicates, the search yielded *n* = 1,747 articles, and the initial screen of abstracts yielded *n* = 745 articles to undergo a full-text review. Ultimately, 55 studies met the eligibility criteria and underwent data extraction and analysis (Figure 1).

55 studies described 55 unique intelligent techniques (Table 2). The studies were analyzed based on the leukaemia type involved in the study, the task, the data source, and the purpose of the study.

### 3.2. Based on Leukaemia Type

**Figure 2:**
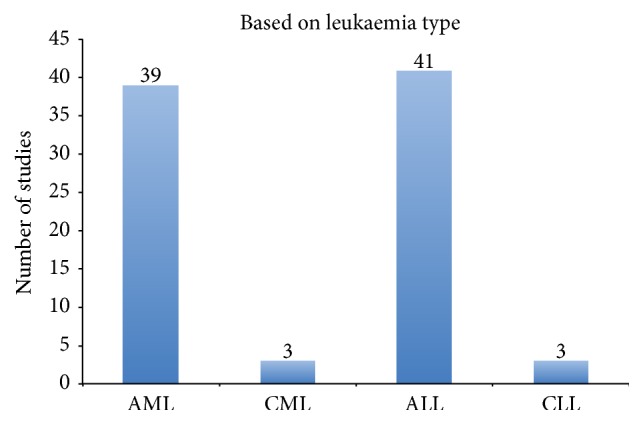
Summary of the frequency of studies based on leukaemia type.

Of the commonest leukaemia types (Figure 2) involved in previous studies, AML and ALL occupied most of the classification studies because the DNA microarray data can be downloaded online from the Cancer Program Legacy Publication Resources [41].

Some studies [42–44] distinguished ALL origin cell lines from non-ALL leukaemia origin cell lines. A study [45] demonstrated a decision support system that classifies all four types of leukaemia using principal components for feature selection and clustering. A few studies involved chronic leukaemia types to identify the molecular biomarker in CML. For example, Oehler et al. [46] used Bayesian model averaging, while Yeung et al. [47] integrated expert knowledge to predict the functional relationships in gene expression. The capture of disease pathophysiology across patient types was studied by Savvopoulos et al. [48], and temporally and spatially distributed models were built to extract knowledge from CLL patients' blood samples. In CML and CLL studies, some studies were not included in the final selection because most of these studies were prospective studies ending with a description of patient population outcomes, rather than building models or using intelligent techniques. A study of combined prognostic markers using a multivariate model for knowledge extraction is included in this study because it proved that integration of gene expression in a model can predict outcomes in CLL [49].

Emphasis has been placed on CML as a research opportunity because of developments in monitoring CML patients' molecular response to molecular targeted therapy. The Australian Institute of Health and Welfare (AIHW) classified myeloid cancers as the 9th most commonly diagnosed cancer in 2016, with around 3,624 cases in Australia [50]. Chronic myeloid leukaemia is also known as chronic myelogenous leukaemia or chronic granulocytic leukaemia. White blood cells are affected when the bone marrow produces an unusual number of white blood cells. These cells enter the bloodstream and accumulate in organs such as the spleen or liver. If the disease progresses, the bone marrow could produce an excessive number of immature white blood cells. Consequently, the bone marrow cannot make enough red cells, normal white cells, and platelets [51].

In CML, according to White and Hughes [52], the previous studies did not establish criteria for selecting the best molecular targeted therapy for each patient, particularly following the availability of multiple therapies that can represent a perfect application of personalized therapy based on predicting patients' molecular response to molecular target therapy.

### 3.3. Based on Data Source

**Figure 3:**
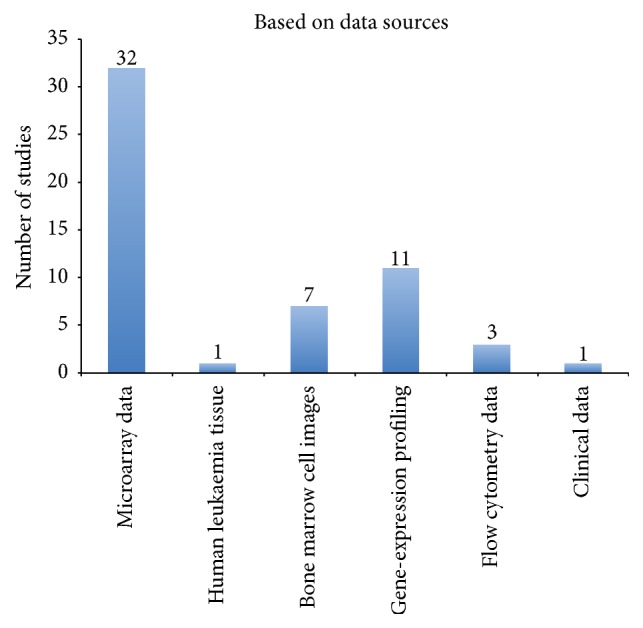
Summary of the frequency of studies based on data sources.

Microarray technology is an area of considerable focus for the purpose of cancer diagnosis (Figure 3). One study [45] used exon arrays to classify patients who suffer from different forms of leukaemia at various stages. Another study used human leukaemia tissue to determine different cluster differentiation (CD) markers [53]. Although seven existing studies used bone marrow cell images to classify leukaemia subtype, microarrays are still used to facilitate different types of experiments and clarify the results, making it easy for researchers to propose molecular medicines in contrast to the rate of tumor progression. The other example used gene-expression profiling among adult patients, which proved crucial in treating leukaemia. The researchers stated that, with the help of expression profiling, doctors are able to determine how a patient adapts to treatment methods. In addition, based on these facts, a physician is able to determine the survival probability of the patients in question.

A huge opportunity arises from integrating data sources such as image data, clinical data, lifestyle or family history, SNP, gene-expression profiles, proteomics profiles, and metabolomics profiles. For example, SNPs have been investigated in an attempt to determine the susceptibility rate of patients suffering from leukaemia, which can support cases where patients have been diagnosed with leukaemia. The use of SNPs made it possible for physicians to predict the likely survivability of their patients after treatment, which is useful in determining the most suitable medical interventions.

In terms of patient care and administration, electronic health records (EHR) are often reused in research to answer specific research questions [5, 54–56]. Cases are matched with enquiries based on obtained research criteria for patient inclusion, and a dataset of many matches can then be generated for analysis. The EHR may include sparse data or missing values, as some patients may not seek frequent care. The quality of EHR would likely impact the bias of research findings or modeling performance. Derivation of key variables is also an important aspect when dealing with EHR, as the values may be recorded in different ways in different systems. This arises due to varying definitions between sources. The quality of data and correct values of derived key variables are of concern to researchers, but many algorithms can be investigated during preprocessing procedures to improve the quality of data, which would possibly lead to more reliable results [57].

Yu et al. [58] also divided the source of data and knowledge into three sources: clinical trial, systems biology, and healthcare systems. The meta-analysis and systematic reviews from published clinical trials are the main sources for gathering data and knowledge, although these methods have limitations over time. The difficulty of refining knowledge as new knowledge arises is an issue, as is the length of time required to build a knowledge base using systematic review and meta-analysis. The second source is system biology. The huge amount of data and knowledge collected as panomics for oncology patients come from genomics, transcriptomics, proteomics, and metabolomics data. For example, the Global Alliance for Genomics and Health [59] provides terabytes of genomic and clinical data for researchers. The third source is healthcare systems that provide knowledge in digital formats.

The other important source that has not attracted much interest in leukaemia studies is the data resulting from clinical trials studying healthy populations or epidemiological studies. Future development of clinical decisions can be guided by lessons learned from previous trials. Late-phase clinical trials (phases II, III, and IV) are considered to be massive sources of information that can be used to build personalized models. There is also a rapid increase in the number of electronic medical research databases that provide an opportunity for researchers to reuse medial data to create mathematical models.

The NCI [60] is a US agency that lists ongoing clinical trials that are testing molecular target therapies, including most of the studies conducted by investigators at hospitals and medical centers. The NCI offers full trial descriptions and names of principal investigators, so researchers can contact investigators and collaborate to conduct the proposed research.

The issue with the clinical trial data that it may be biased in several aspects: sampling, referral, selection, method, and clinical spectrum biases. Clinical trials may use sampling methods, sample size, and inclusion and exclusion criteria. Another aspect is referral bias where patients are referred by specialists and the data will represent preselected patients who have high prevalence of disease. Selection bias is clear when the clinical trial data includes groups based on a variety of demographics. In the method aspect, the data may be collected using different measurements, which leads to varying precision and specifications. Finally, the clinical spectrum bias represented in patient records may show other medical problems apart from the disease [61]. For instance, Saussele and Pfirrmann [62] have reported clinical trials in CML. They demonstrated several aspects that may cause challenges to reusing clinical trials. According to Saussele and Pfirrmann, the definition of “remission” varies in clinical trials by major molecular response (MMR) or complete cytogenetic response (CCR). In addition, the clinical trials used different primary endpoints such as 12 months' MMR or 12 months' CCR to judge treatment success. The American Society of Hematology (ASH) has established annual meetings to discuss medical trial outcomes. In 2006, the 48th ASH Annual Meeting [63] displayed a poster about the International Randomized Study of Interferon versus Imatinib STI571 (IRIS) trial. This study showed that Imatinib is appropriate for CML patients in the chronic phase as results indicate that patients receiving long-term (5-6 years) Imatinib therapy achieve MMR in 90% of cases [64]. For CML patients, these results show the efficacy of continuing to receive Imatinib over time. In 2010, two significant medical trials, DASISION (Dasatinib versus Imatinib Study in Treatment-Naïve CML) and ENESTnd (Evaluating Nilotinib Efficacy and Safety in Clinical Trials-Newly Diagnosed Patients), were discussed at the 52nd Annual Meeting [65]. In DASISION [66], 259 patients were studied for their response to 100 mg Dasatinib once daily versus 260 patients who consumed 400 mg of Imatinib once daily. The MMR was lower for the Imatinib set compared to the Dasatinib set. The most important result was the safety profiles of these drugs, which were similar. The ENESTnd trial focused on the comparison of Nilotinib versus Imatinib [67]. The samples were newly diagnosed CML-CP patients. In the trial, 282 patients were given 300 mg Nilotinib twice daily, 281 patients received 400 mg Nilotinib once daily, and 283 patients received 400 mg Imatinib once daily; the median follow-up for these patients was 18 months. The cytogenetic response (CCyR) and MMR were lower in patients treated with Imatinib than in patients treated with Nilotinib. From these trial results, the debate about selecting the optimal TKI to achieve optimal patient response has been established with recognition of the need for a predictive assay that could predict the patient's response to initial treatment. The researchers also recognize the need for a medical trial to compare each TKI against the others as a first-generation treatment. The introduction of molecular targeted drugs (TKIs) has led to a dramatic improvement in the lifespan of patients affected by this condition. With the three common TKIs, Imatinib, Nilotinib, and Dasatinib currently approved to use as frontline therapy, an important question arises regarding which TKI should be prescribed. White and Hughes [52] stated that, with the lack of clear recommendations about which TKI to select, clinicians may prescribe a particular TKI based on their own preference. It is possible to extract knowledge and modeling systems using medical data and knowledge in leukaemia, but it requires advanced computational methods, such as intelligent systems. Using the available data and knowledge to construct a personalized medicine support system for leukaemia may provide massive amounts of information to use for evaluating therapies and also for potential diagnostic and prognostic markers.

### 3.4. Based on the Purpose of the Studies

Medical research studies have several purposes, including classification of cancer types or distinguishing healthy cells from unhealthy cells for the purpose of diagnosis, identifying markers to help in the management of treatment, and determining the prognosis of risk. Managing leukaemia patients has gained attention since a successful study by Alvey et al. [68], who developed an expert system by using a tree-structured logical program and produced over 700 clinical diagnostic rules. Even though this study focused on a specific diagnostic aspect of the system, another study [69] provided information for managing patients from registration to diagnosis and through follow-up after treatment. The study integrated history, physical examinations, and laboratory data to develop a decision support system for leukaemia diagnostics. Chae et al. [69] used profiles from patients admitted to Severance Hospital in Seodaemun District, Republic of Korea, and used data from over 490 patients to discover knowledge that helped physicians in decision-making. There is some evidence that interdisciplinary cooperation between biologists, medical scientists, computer scientists, and engineers can be productive. However, this research team did not incorporate molecular data in their system.

**Figure 4:**
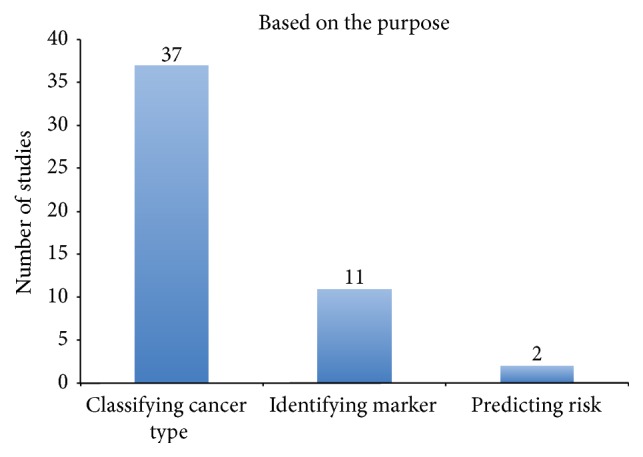
Summary of the frequency of studies based on the purpose of the studies.

Among the 55 studies (Figure 4), the common purpose for conducting the studies was classifying the cancer type or subtype, with the aim of diagnosing leukaemia patients. An identifying marker in support of treatment management was observed in 11 studies. The other purpose for conducting the studies was using intelligent techniques and statistical methods for prognosis [70–73]. For example, predicting a relapse prior to transplantation in CML by integrating iterative Bayesian model averaging includes expert knowledge and expected functional connections in expression analyses in order to recognize genes causative of CML evolution [47]. This kind of model provides high-quality results, especially in complex diseases, but has varying levels of classification precision.

A new development is to extract relationships between biomarkers and the outcome in leukaemia patients. Focusing on CML, a predictive factor is a patient characteristic used to predict response to treatment [74]. The predictive factors related to the MMR response include common molecular assays. Other factors depend on peripheral blood counts as well as on the molecular-based and clinical observations of individual patients. In order to select the most effective TKI therapy at the time of diagnosis, various predictive factors in CML have been investigated to distinguish patients at an increased risk of failure with Imatinib, the first-generation TKI [52, 75–77].  Table 1 shows the current predictive assays and score systems, the factors included in the score systems and the methods used, the target prediction, and the published results.

Many studies [77, 129] have shown that predictive factors could probably assist in predicting patient response. Milojkovic et al. [129] conducted their study in order to predict success or failure of treatment with second-generation TKIs in CML patients using univariate analyses. They analyzed a cohort of 80 CML patients in the first chronic phase who were treated with Dasatinib or Nilotinib. Their score system predicted the probability of CML patients achieving a CCR. The system was based on three factors: cytogenetic response to Imatinib, Sokal score, and recurrent neutropenia during Imatinib treatment. Although this study used simple statistical methods, the system succeeded in classifying three risk categories: good, intermediate, and poor risk. In addition, Jabbour et al. [77] also studied the factors for predicting 123 CML patients' response after Imatinib failure. The variables used in this study included sex, CML duration, months' performance status, splenomegaly, prior interferon therapy, peripheral blood, bone marrow, best cytogenetic response to Imatinib, second-generation Nilotinib or Dasatinib therapy, active disease at the start of the second course of TKIs, clonal evaluation, higher than 90% Ph positivity, and IC50 for Nilotinib and Dasatinib for in vitro inhibition of kinase activity of the mutated point in* BCR-ABL*. They also used univariate and multivariate analyses, such as the logistic regression model and the Cox proportional hazard model, in order to identify prognostic factors associated with MCyR and survival, and they succeeded in identifying three risk groups: low, intermediate, and high risk.

Previously, two of the predictive factors closely involved in predicting the molecular response in CML were identified. The first such factor is IC50. In 2005, White et al. [130] studied inhibitory concentration 50% (IC50^imatinib^) as a predictor of molecular response for CML patients. The results demonstrate that IC50^imatinib^ is a powerful pretreatment predictor [131]. The second factor is the activity of organic cation transporter 1 (OCT-1). There are two functions for OCT proteins, which are cellular uptake and excretion of a number of exogenous and endogenous cationic and uncharged substances. The OCT-1 protein activity (OA) can be measured by uptake in the presence and absence of a specific OCT-1 inhibitor. It has been found that patients with high OA have a better molecular response than patients with low OA; therefore, OA is considered a predictive factor for response to Imatinib, but not for Nilotinib or Dasatinib [131, 132]. White et al. propose [133] that, in CML patients treated with Imatinib, the use of OA pretherapy was a predictor for long-term resistance risk and could be used to individualize dosage strategies. Thus, involving OA to estimate the response could lead to better results, but only for Imatinib therapy. A recent study has investigated the possible association between molecular response and a number of factors such as Sokal score, age, sex, and Imatinib dose [134]. It was also found that being female is a strong predictor [134]. A recent review of biomarkers that determine prognosis in CML also presented a list of prognostic indicators at diagnosis, such as the three scoring systems,* BCR-ABL1* transcript type, and OA [135]. Another factor is the* BCR-ABL* transcript type; CML patients with the b3a2* BCR-ABL1* transcript type, compared to those with the b2a2 transcript type, demonstrate greater survival rates, while CML patients with the p190 transcript type are classified as high risk [136, 137].

In practice, clinicians aim to treat individual CML patients with the most beneficial therapy. This can be made possible by using accurate risk assessment methods at diagnosis. When there is any doubt about either the diagnosis or the recommended treatment, a second opinion is often sought before considering any treatment. The need for multiple prognostic scores can occur frequently in a complex problem that has multiple independent experts with varying expertise. When developing prognostic scores have different patient populations, each score can capture different knowledge. There are two general major objectives for combining prognostic scores: first, one prognostic score enhances the decision of another one; and second, it increases the reliability of the final decision. However, integrating multiple prognostic scores could generate conflict in decisions and may not be sufficient to make a final decision.

It is important that clinicians are comfortable with a wide range of prognostic scores that will help to identify risk category because a conflict between scores may be observed in some patients. Consistency is defined as a score that does not contradict other prognostic scores. Consistency among prognostic scores can increase clinicians' trust, as they rely on such results to make appropriate treatment decisions. It is important to study and understand the consistency of scores to help clinicians categorize patients into suitable risk groups and subsequently make better therapeutic decisions.

In light of the aforementioned aspects, it is necessary to conduct a study that can contribute to the CML medical field by solving the previous issues. Using machine-learning techniques and fusion techniques to address these problems could produce promising results. The first proposed solution is to build a personalized medicine support system as a predictive model to combine strong molecular, clinical data, and predictive assays for CML patients that could probably predict an individual molecular response. Moreover, predicting an individual response leads to predicting warning groups for each TKI. From a computer-science perspective, the above issues could be resolved by using a machine-learning algorithm that combines the most effective predictive indicators to predict the outcomes for each TKI, based on existing clinical profiles for individual CML patient characteristics. The main goal of this review is to improve the ability to manage CML disease in individual CML patients. Therefore, CML is an example of a research opportunity to predict the molecular response to TKI treatment. Using intelligent computing techniques could bring about promising results for CML patients.

### 3.5. Based on the Task

**Figure 5:**
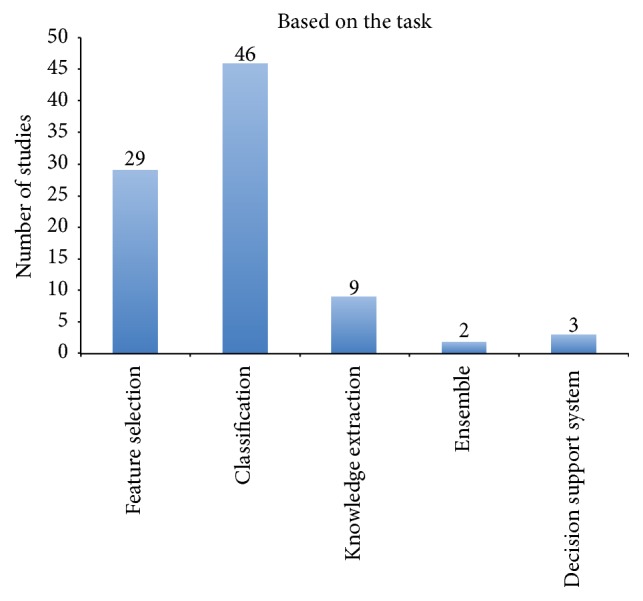
Summary of the frequency of studies based on the task.

Most of the studies that used machine learning and data mining incorporated two major tasks: feature selection and classification (Figure 5). Although dissimilar feature-selection algorithms may possibly choose dissimilar pertinent genes or diverse numbers of relevant genes or bring about different levels of classification precision [138], feature selection can utilize observations and functions to obtain dimensions for exploring optimal solutions [139]. In addition, selection of a subset in a classifier reduces the computational time and costs of study, thereby increasing classification accuracy.

Many studies [53, 89, 91, 103, 105, 112, 118] used classification algorithms without feature-selection techniques. Since cancer tumors are highly diverse in their genetic patterns and progressions, DNA arrays provide a platform to obtain the best measurements and observations, helping assign objectives to one relevant feature set and hence contributing to precise convergence toward optimal results [140]. However, some researchers [42–44, 82, 86, 87, 94, 96–99, 104, 108, 113, 115, 122, 141, 142] applied two common methods in feature selection: filter and wrapper approaches with classification algorithms. Considering feature-selection techniques is an essential preprocessing method mandated for classification processes [103].

Knowledge extraction or acquisition has been a great challenge for researchers, as they exhibit unusual characteristics in many different genes relative to the number of tumor samples. AML acquires a similar appearance to ALL, which makes it nearly impossible for researchers to distinguish between synonymous patterns. However, Cho et al. [143] proposed an approach to form the optimal linear classifier by means of gene-expression data. They used discriminant partial least squares and linear discriminant analysis to differentiate between acute leukaemia subtypes. They found that these methods offered a satisfactory level of precision. They concluded that the suggested method builds the optimal classifier made up of a highly accurate, small-size predictor.

Using multiple algorithms for knowledge extraction and classification has not attracted much interest from leukaemia researchers in previous studies [45, 140, 144]. Cho and Won [86] were of the view that conventional machine learning is incapable of delivering accurate information. For this reason, they developed a novel ensemble machine-learning approach for microarray classification. Results indicated that the ensemble machine-learning approaches accuracy of almost 97% in leukaemia classification, which makes it a better alternative to basic machine-learning methods.

Among the 55 studies, three groups of researchers [45, 121, 140] built decision support systems, whose subfunctions included multiple tasks. The first decision support system [45] was built to support leukaemia diagnosis using exon array analysis. The system combined intelligent techniques, such as preprocessing and data-filtering techniques, clustering for classifying patients, and extraction of knowledge techniques. The authors suggested that further study of bone marrow or blood samples may assist in diagnosis of leukaemia stages. The second study [140] was conducted to extract decision rules using a developed SVM. This study comprised carrying out multiple tasks, including data fusion, feature selection, making a prediction model based on gene-expression data, and knowledge extraction. The third study [121] involved developing decision support to identify unhealthy ALL cells using feature-selection techniques with SVM.

From the review of studies based on the task, the need for personalized medicine in CML results in multiple active TKI therapies as molecular targeted therapy available for CML, multiple strategies utilized for frontline CML therapy, heterogeneity in responses, and multiple prognostic scores and predictive assays.

Therapy takes the form of two major strategies: (i) frontline Imatinib or (ii) frontline second-generation TKIs such as Nilotinib or Dasatinib [4]. Despite the remarkable increase in the survival of CML patients treated with Imatinib, some patients discontinue Imatinib therapy due to intolerance, resistance, or progression. In the IRIS [64] trial, it is demonstrated that variations in molecular response at 12 and 18 months of Imatinib was due to, in about 40% of cases, discontinuing Imatinib because of intolerance or resistance and due to further progression observed in 7% of CML patients.

Hematologic, cytogenetic, and molecular strategies for monitoring patient responses to therapies are used by European LeukaemiaNet [145]. To monitor molecular response, RQ-PCR, which is a sensitive technique, is used to quantify the level of* BCR-ABL1* mRNA transcripts in the peripheral blood of patients. Molecular monitoring is considered to be a standard guide to clinical management in CML [146, 147]. The prediction of long-term molecular response to frontline Imatinib in CML can help clinicians to select the best treatment protocols for CML patients. Patients predicted not to achieve MMR in the long term might be better treated with alternative frontline therapies, such as Nilotinib or Dasatinib. Opportunities to improve individual care for CML patients exist in the appropriate prediction of variation in treatment response to support physicians in treatment decisions.

Prognostic scores are used to personalize CML patient care by predicting responses to therapy. Although the prognostic scores (Sokal, Hasford, EUTOS, and the ELTS scores) remain in use today, they were developed either for identifying risk groups or for predicting cytogenetic response to therapy, but not for molecular response. Although two predictive assays, IC50^imatinib^ and OCT-1 activity (OA), were developed to predict molecular response, according to current knowledge, a model using assays to predict molecular response has not previously been considered. The combination of predictive assays results in greater predictive power than that which each predictor provides alone [148, 149].

## 4. Conclusion

Modern oncology is experiencing a paradigm shift toward personalized medicine, which aims to direct medical agents toward the tumor site. The field of molecular medicine is also undergoing transformational changes that are bringing a much needed revolution in healthcare. This breakthrough was made possible by technologies in genetic studies that led to the sequencing of the human genome. An analysis of biological samples from whole organisms has now been made possible. In addition, this invention has given a new lease of life to the treatment of cancer. However, the majority of cancer patients have been shown to develop adverse drug reactions due to overreliance on certain medications.

Intelligent techniques may be useful for clinicians in decision-making, warning of specific problems or providing treatment recommendations [150]. In that regard, it would be worthwhile building personalized medicine support system to work as predictive models that integrate molecular-based data to predict cancer susceptibility, including risk assessment, prediction of the probability of developing a type of cancer prior to occurrence of the disease, prediction of recurring cancer, and the prediction of cancer outcomes, such as survivability, life expectancy, and response to therapy or progression. This is highly advantageous since only a quarter of cancer patients respond positively to the drugs prescribed to them. Therefore, it is important to investigate the current development of using molecular information in intelligent models for personalized medicine.

The use of personalized medicine support systems in medicine will bring a ray of hope to the treatment of leukaemia. Other frontiers of personalized medicine research, such as the role of genetics in infectious diseases, proteomics, epigenetics, and metabolomics, were not covered by this review and are out of scope of this research. This review was conducted based on current developments of personalized medicine support systems, and a systematized literature review was carried out on intelligent techniques using molecular data analysis in leukaemia. Both sets of literature led to identifying opportunities for further research for personalized medicine support systems in one category of leukaemia, namely, chronic myeloid leukaemia. We speculate that this paper will assist health informatics and biomedical and bioinformatics in order to answer specific technical questions to help develop future research into leukaemia from a technical perspective.

## Figures and Tables

**Table 1:** The current methods used to identify risk in CML.

Previous methods				
Study	Factors	Method	Target prediction	Data and results
Sokal score, Sokal et al. [78]	Age, spleen size (cm), blast (%), and platelets (10^9^/L)	Multivariate analysis of survival	Risk groups for chemotherapy	Six European and American sources (*n* = 813), low 39%, intermediate 38%, and high 23%

Hasford score, Hasford et al. [79]	Age, spleen size (cm), blasts (%), eosinophils (%), basophils (%), and platelets (10^9^/L)	Multivariate analysis of survival	Risk groups for interferon alpha alone	14 studies (*n* = 981), low 40.6%, intermediate 44.7%, and high 14.6%

EUropean Treatment Outcome Study (EUTOS) Score, Hasford et al. [80]	Basophils (%) and spleen size (cm)	Multivariate analysis of response	CCgR at 18 months to Imatinib	Five national study groups (*n* = 2,060), low 79% and high 21%

EUTOS Long-Term survival (ELTS) score, Hoffmann et al. [81]	Age, spleen size (cm), blast (%), and platelets (10^9^/L)	Multivariate analysis of response	Long-term survival	(*n* = 2,205) low 61%, intermediate 27%, and high 12%

**Table 2:** Review of the studies, data sources, their purpose, and machine-learning algorithms reported from 2001 to 2015.

	Study	Year	Tasks	Data source	Leukaemia types involved in the study	Purpose	Methods
1	Cho [82]	2002	Feature selection and classification	DNA microarray	AML, ALL	Classifying leukaemia types	Pearson's and Spearman's correlation coefficients, Euclidean distance, cosine coefficient, information gain, mutual information and signal-to-noise ratio being used for feature selection

2	Inza et al. [83]	2002	Feature selection and classification	DNA microarray	AML, ALL	Classifying cancer, select genes related to cancer	Feature subset selection, case-based, and nearest neighbor classifier

3	Farag [84]	2003	Feature selection and classification	Blood cells image	AML, ALL	Classifying leukaemia types	A three-layer backpropagation neural network

4	Futschik et al. [85]	2003	Knowledge discovery	Gene expression	AML, ALL	Classifying leukaemia types and select gene expression	Knowledge-based neural networks and evolving fuzzy neural networks and adaptive learning and rule extraction

5	Cho and Won [86]	2003	Feature selection, classification, and ensemble classifiers	DNA microarray	AML, ALL	Classifying leukaemia types and select genes related to cancer	Correlation coefficient, Euclidean distance, cosine coefficient, information gain, mutual information, a feed-forward multilayer perceptron, *k*-nearest neighbor, self-organizing map, and support vector machine. Majority voting, weighted voting, and Bayesian approach

6	Marx et al. [44]	2003	Feature selection and classification	DNA microarray	AML, ALL	Classifying leukaemia from nonleukaemia	Principal component analysis and clustering

7	Marohnic et al. [87]	2004	Feature selection and classification	DNA microarray	AML, ALL	Classifying leukaemia types	Mutual information and support vector machine

8	McCarthy et al. [88]	2004	Knowledge extraction, classification, feature selection, visualization	Proteomic mass spectroscopy data, and gene expression	Melanoma, leukaemia	Cancer detection, diagnosis, and management	Naïve Bayes, support vector machines, instance-based learning (*K*-nearest neighbor), logistic regression, and neural networks

9	Rowland [89]	2004	Classification	Gene expression	AML, ALL	Classifying leukaemia types	Genetic Programming

10	Markiewicz et al. [90]	2005	Feature selection and classification	Images of different blast cell	Myelogenous leukaemia	Classifying patients	Support vector machine

11	Tung and Quek [91]	2005	Classification	DNA microarrays	ALL	Classifying leukaemia types	A neural fuzzy system, NN, SVM and the *K*-nearest neighbor (*K*-NN) classifier

12	Nguyen et al. [92]	2005	Classification	DNA microarrays	AML, ALL	Classifying leukaemia types	Support vector machine (SVM)

13	Plagianakos et al. [93]	2005	Feature selection and classification	DNA microarrays	AML, ALL	Classifying leukaemia types	artificial neural networks

14	Li and Yang [94]	2005	Feature selection and classification	DNA microarrays	AML, ALL	Classifying leukaemia types	SVM, ridge regression and Rocchio, feature selection in recursive and nonrecursive settings

15	Jinlian et al. [95]	2005	Knowledge extraction	DNA microarray	AML, ALL	Leukaemia gene association structure	Clusters

16	Diaz et al. [96]	2006	Feature selection and classification	DNA microarrays	Acute Promyelocytic Leukaemia	Classifying Acute Promyelocytic Leukaemia (APL) from the non-APL leukaemia	Discriminant fuzzy pattern

17	Feng and Lipo [97]	2006	Feature selection and classification	DNA microarrays	AML, ALL	Acute leukaemia types	*t*-statistics to rank the gene and support vector machines

18	Nguyen and Ohn [98]	2006	Feature selection and classification	DNA microarrays	AML, ALL	Classifying leukaemia types	Dynamic recursive feature elimination and random forest

19	Shulin et al. [99]	2006	Feature selection and classification	DNA microarrays	AML, ALL	Classifying leukaemia types	Independent component analysis and SVM

20	Chen et al. [100]	2007	Feature selection, rule extraction, and classification	DNA microarrays	AML, ALL	Classifying leukaemia types	A multiple kernel support vector machine

21	Ujwal et al. [43]	2007	Feature selection and classification	DNA microarray	ALL	Identifying functional cancer cell line classes, classifying leukaemia from nonleukaemia	*p* value and clustering

22	Perez et al. [101]	2008	Classification	Gene expression	AML, ALL	Classify leukaemia types	Hybrid fuzzy-SVM

23	Yoo and Gernaey [42]	2008	Feature selection and classification	DNA microarrays data	ALL	Classifying ALL origin cell lines from non-ALL leukaemia origin cell lines	Discriminant partial least squares, principal component and Fisher's linear discriminant analysis, linear discriminant function and SVM, and hierarchical clustering method

24	Avogadri et al. [102]	2009	Knowledge extraction	Gene expression	Myeloid leukaemia	Discovering significant clusters	Stability-based methods

25	Eisele et al. [49]	2009	Knowledge extraction	Gene expression	CLL	Prognostic markers	Multivariate model

26	Chaiboonchoe et al. [103]	2009	Classification	DNA microarrays data	ALL	Identification of differentially expressed genes	Self-organizing maps (neural networks), emergent self-organizing maps (extension of neural networks), the short-time series expression miner (STEM), and fuzzy clustering by local approximation of membership (FLAME)

27	Oehler et al. [46]	2009	Knowledge extraction	Gene expression	CML	Identifying molecular markers	Bayesian model averaging

28	Corchado et al. [45]	2009	Decision support system preprocessing, filtering, classification, and extraction of knowledge	Exon arrays	ALL, AML, CLL, CML	Classifying patients who suffer from different forms of leukaemia at various stages	Principal components, clustering, CART

29	Glez-Peña et al. [104]	2009	Feature selection and classification	DNA microarray	AML	Classifying gene expression	Fuzzy pattern algorithm

30	He and Hui [105]	2009	Classification	DNA microarray	ALL, AML	Classifying leukaemia types	Ant-based clustering (Ant-C) and an ant-based association rule mining (Ant-ARM) algorithms

31	Mukhopadhyay et al. [106]	2009	Feature selection and classification	DNA microarray	ALL, AML	Classifying leukaemia types	GA-based fuzzy clustering, neural network, and support vector machine

32	Torkaman et al. [107]	2009	Classification	Human leukaemia tissue	ALL, AML	Determining different CD markers	Cooperative game

33	Zheng et al. [108]	2009	Feature selection	DNA microarray	ALL	Gene ranking	Knowledge-oriented gene selection

34	Mehdi et al. [109]	2009	Knowledge acquisition	Gene expression	ALL, AML	Pattern clustering	*K*-nearest neighbor technique

35	Porzelius et al. [110]	2011	Feature selection, classification	Microarray and clinical data	ALL	Risk prediction	Feature selection approach for support vector machines as well as a boosting approach for regression models

36	Chen et al. [111]	2011	Feature selection, data fusion, class prediction, decision rule extraction, associated rule extraction, and subclass discovery	DNA microarray	ALL, AML	Select gene, classify leukaemia types, rule extraction	Multiple kernel SVM

37	Gonzalez et al. [112]	2011	Classification	Bone marrow cells images	ALL, AML	Classifying leukaemia subtypes	Segmentation method to obtain leukaemia cells and extract from them descriptive characteristics (geometrical, texture, statistical) and eigenvalues

38	Tong and Schierz [113]	2011	Feature selection and classification	DNA microarray	ALL, AML	Classifying two-class oligonucleotide microarray data for acute leukaemia	Hybrid genetic algorithm-neural network

39	Chauhan et al. [114]	2012	Classification	Genotype	ALL, AML	Identifying gene-gene interaction	Classification and regression tree

40	Escalante et al. [115]	2012	Feature selection and classification	The morphological properties of bone marrow images	ALL, AML	Classifying leukaemia subtypes	Ensemble particle swarm model selection

41	Yeung et al. [116]	2012	Feature selection and classification	Gene expression	CML	select gene, and predicted functional relationships	Integrating gene expression data with expert knowledge and predicted functional relationships using iterative Bayesian model averaging

42	Manninen et al. [117]	2013	Classification	Flow cytometry data	AML	Prediction method for diagnosis of AML	Sparse logistic regression

43	El-Nasser et al. [118]	2014	Classification	DNA microarrays	ALL, AML	Classifying leukaemia types	Implement enhanced classification (ECA) algorithm, SMIG module, and ranking procedure.

44	Singhal and Singh [119]	2015	Feature selection and classification	Image based analysis of bone marrow samples	ALL	Classifying leukaemia subtypes	Multilayer perceptron (MLP), linear vector quantization (LVQ), *k*-nearest neighbor (*k*-NN), and SVM

45	Yao et al. [120]	2015	Feature selection and classification	DNA microarrays	ALL, AML, the mixed-lineage leukaemia (MLL) data	Classifying leukaemia subtypes	Random forests and ranking features

46	Rawat et al. [121]	2015	Computer-aided diagnostic system, feature selection, and classification	Bone marrow cells in microscopic images	ALL	Diagnosis lymphoblast cells from healthy lymphocytes	Support vector machine

47	Kar et al. [122]	2015	Feature selection and classification	DNA microarrays	ALL, AML, the mixed-lineage leukaemia (MLL) data	Classifying leukaemia subtypes	Particle swarm optimization (PSO) method along with adaptive *K*-nearest neighborhood (KNN)

48	Li et al. [123]	2016	Classification	Gene expression	AML	Identifying feature genes	Support vector machine (SVM) and random forest (RF)

49	Dwivedi et al. [124]	2016	Classification	Microarray gene expression	ALL, AML	Classifying leukaemia subtypes	Artificial neural network (ANN)

50	Krappe et al. [125]	2016	Classification	Image based analysis of bone marrow samples	Leukaemia	Diagnosis of leukaemia and classifying 16 different classes for bone marrow	Knowledge-based hierarchical tree classifier

51	Li et al. [123]	2016	Classification	DNA microarrays	AML, ALL	Classifying leukaemia subtypes	A weighted doubly regularized support vector machine

52	Ocampo-Vega et al. [126]	2016	Feature selection and classification	DNA microarrays	AML, ALL	Classifying leukaemia subtypes	Principal component analysis and logistic regression

53	Rajwa et al. [127]	2016	Classification	Flow cytometry data	AML	Determining progression of the disease	Nonparametric Bayesian framework

54	Ni et al. [128]	2016	Classification	Flow cytometry data	AML	Analyzing minimal residual disease	Support vector machines (SVM)

55	Savvopoulos et al. [48]	2016	Knowledge extraction	CLL cells in peripheral blood	CLL	Capturing disease pathophysiology across patient types	Temporally and spatially distributed model
