# Corrigendum to “Intelligent Techniques Using Molecular Data Analysis in Leukaemia: An Opportunity for Personalized Medicine Support System”

**DOI:** 10.1155/2018/8208254

**Published:** 2018-06-21

**Authors:** Haneen Banjar, David Adelson, Fred Brown, Naeem Chaudhri

**Affiliations:** ^1^Department of Computer Science, King Abdulaziz University, Jeddah, Saudi Arabia; ^2^School of Computer Science, University of Adelaide, Adelaide, SA, Australia; ^3^School of Molecular and Biomedical Science, University of Adelaide, Adelaide, SA, Australia; ^4^Oncology Centre, Section of Hematology, HSCT, King Faisal Specialist Hospital and Research Centre, Riyadh, Saudi Arabia

In the article titled “Intelligent Techniques Using Molecular Data Analysis in Leukaemia: An Opportunity for Personalized Medicine Support System” [1], there was an error in the order of the first and second affiliations. The corrected affiliations' list is shown above.
